# Influence of Massage and Skin Hydration on Dermal Penetration Efficacy of Nile Red from Petroleum Jelly—An Unexpected Outcome

**DOI:** 10.3390/pharmaceutics13122190

**Published:** 2021-12-18

**Authors:** Vasudha Kaushik, Yameera Ganashalingam, Robert Schesny, Christian Raab, Soma Sengupta, Cornelia M. Keck

**Affiliations:** Department of Pharmaceutics and Biopharmaceutics, Philipps-Universität Marburg, Robert-Koch-Str. 4, 35037 Marburg, Germany; vasudha.kaushik@pharmazie.uni-marburg.de (V.K.); yameera.ganashalingam@pharmazie.uni-marburg.de (Y.G.); robert.schesny@pharmazie.uni-marburg.de (R.S.); christian.raab@pharmazie.uni-marburg.de (C.R.); sengupts@pharmazie.uni-marburg.de (S.S.)

**Keywords:** skin, dermal penetration, Vaseline, paraffin, solvent drag, massage, hydration, ex vivo, TEWL

## Abstract

The study aimed at comparing the influence of direct and indirect skin hydration as well as massage on the dermal penetration efficacy of active compounds. Nile red was used as a lipophilic drug surrogate and was incorporated into Vaseline (petroleum jelly). The formulation was applied with and without massage onto either dry skin or pre-hydrated, moist skin. It was expected that the occlusive properties of Vaseline in combination with massage and enhanced skin hydration would cause a superposition of penetration-enhancing effects, which should lead to a tremendous increase in the dermal penetration efficacy of the lipophilic drug surrogate. Results obtained were diametral to the expectations, and various reasons were identified for causing the effect observed. Firstly, it was found that Vaseline undergoes syneresis after topical application. The expulsed mineral oil forms a film on top of the skin, and parts of it penetrate into the skin. The lipophilic drug surrogate, which is dissolved in the mineral oil, enters the skin with the mineral oil, i.e., via a solvent drag mechanism. Secondly, it was found that massage squeezes the skin and causes the expulsion of water from deeper layers of the SC. The expulsed water can act as a water barrier that prevents the penetration of lipophilic compounds and promotes the penetration of hydrophilic compounds. Based on the data, it is concluded that dermal penetration is a complex process that cannot only be explained by Fick’s law. It is composed of at least three different mechanisms. The first mechanism is the penetration of active ingredients with their solvents into the skin (convection, solvent drag), the second mechanism is the penetration of the active ingredient via passive diffusion, and the third mechanism can involve local penetration phenomena, e.g., the formation of liquid menisci and particle-associated penetration enhancement, which occur upon the evaporation of water and/or other ingredients from the formulation on top of the skin.

## 1. Introduction

Dermal drug delivery is an important administration route and can be used for the local and systemic administration of active ingredients (AI). In most cases, topical products that allow for the effective dermal or transdermal penetration of the AI are desired. The penetration efficacy is known to be influenced by various parameters, i.e., the type of AI (polarity, molecular weight, etc.), the type of vehicle, type of skin, and type of application (for example, with or without massage) [1,2,3,4]. Today, different in vitro, ex vivo, and in vivo methods are available for the testing of the dermal penetration efficacy, where the most often used method is in vitro testing with Franz diffusion cells [5]. Testing the dermal penetration with Franz diffusion cells is tedious work and requires experienced and careful handling. Recent results, however, now demonstrated that results obtained by this technique can even be misleading if poorly soluble and non-dissolved active compounds are tested [6]. The reason for this is that the recommended test setup of Franz diffusion cells requires an acceptor medium that provides sink conditions for the test compound. For a correct test setup, the acceptor medium needs to be in close contact with the test membrane to allow for a non-hindered diffusion of the active compounds through the membrane into the acceptor medium. Recent studies have now demonstrated that the acceptor medium can also penetrate into the test membrane [6]. In these cases, the acceptor medium is soaked in the test membrane, and thus the formulation that is applied onto the test membrane or skin will also come into direct contact with the acceptor medium. If non-dissolved active compounds come into contact with this acceptor medium-soaked membrane, they will dissolve and quickly penetrate through the test membrane/skin, leading to results that suggest good permeability of the test compound [6]. In a physiological setup, i.e., skin in its original state, the skin is not soaked with solvents and thus non-dissolved active compounds will possess different dissolution behaviors than in the Franz diffusion setup. Recent tests showed opposite results in penetration efficacy for suspensions that contained microparticles or nanocrystals when compared to a patch in which the active compound was loaded in amorphous state [6]. When tested in vitro with Franz diffusion cells, the suspension that contained the AI in a non-dissolved state as microcrystals led to the highest penetration efficacy, and the patch resulted in the lowest penetration. In contrast, the lowest penetration efficacy was found for the microparticle suspension, and the best penetration for the patch when the formulations were tested with the ex vivo porcine ear model with subsequent digital image analysis. Based on these findings, it was concluded that the use of the ex vivo porcine ear model with subsequent digital image analysis is a more suitable model for testing the dermal penetration efficacy of active compounds from topical formulations [6].

A further advantage of the ex vivo penetration model is its ability to determine changes in the stratum corneum thickness and other skin conditions (for example, transepidermal water loss, water content of the skin, skin friction, skin pH, etc.). This means that the influence of the formulation on the skin properties can also be determined. With this, the ex vivo porcine ear model represents a highly sensitive model that allows for the determination of the penetration efficacy of chemical compounds and the detection of changes in skin conditions that occur upon treatment of the skin with the topical formulation. This combination therefore enables a more holistic view of the performance of topical formulations and should allow for the development of more effective topical products, i.e., products that provide excellent penetration for active compounds and skin-caring properties at the same time [7,8,9].

As the ex vivo porcine ear model is able to detect changes in skin hydration, it was hypothesized that it might also be useful to investigate to what extent skin hydration influences the dermal penetration efficacy of active compounds. Skin hydration can be achieved by a direct hydration of the skin, i.e., by wetting the skin with water and/or by the application of hydrogels or other water-rich vehicles. In addition, skin hydration can also be increased by indirect hydration, i.e., by the application of occlusive formulations that moist the skin by preventing the evaporation of water from the skin. As high skin hydration is considered to cause better dermal penetration, it was speculated that the combination of direct hydration and indirect hydration by occlusion might lead to a “super hydration” of the skin, which might result in a super-additive penetration efficacy. Moreover, it was speculated that massage (rubbing of the skin), which is often considered to foster penetration efficacy [10], might be able to further improve the penetration efficacy. The aim of this study was to prove or to disprove these theories.

Vaseline was used as vehicle in this study. It is an isogel that consists of a complex mixture of liquid and solid hydrocarbons. The solid hydrocarbons are considered to form a three-dimensional crystalline network in which the liquid hydrocarbons are incorporated. Vaseline is also known as petroleum jelly and is an often-used vehicle for the formulation of dermal drug products. It can be used as a vehicle for active pharmaceutical compounds without further excipients and/or as a base for the formulation of various creams and ointments. Vaseline has now been available for 150 years, and many studies have been performed to understand the special properties and manifold applications and mechanisms of action after the topical application of this important pharmaceutical excipient [11,12]. Vaseline is a very cost-efficient excipient, and thus it is an attractive ingredient for topical products [13]. The major properties of Vaseline include its excellent spreading properties on skin and its highly occlusive properties, which are associated with improved dermal penetration efficacy of active ingredients (AI). Nile red (NR) is a lipophilic fluorescent dye and was used as surrogate for an active pharmaceutical or cosmetic ingredient.

## 2. Materials and Methods

### 2.1. Materials

Vaseline and liquid paraffin were obtained from Caesar & Loretz GmbH, Hilden, Germany. Nile red was purchased from Sigma-Aldrich Chemie GmbH (Steinheim, Germany). In addition, in the second part of the study, a water-soluble, fluorescent dye (sodium-fluorescein, Carl Roth GmbH, Karlsruhe, Germany) was used to substantiate the findings of the first part of the study. Purified water for direct skin hydration was obtained from a PURELAB^®^ Flex 2 water purification system (ELGA Labwater, Veolia Water Technologies Deutschland GmbH, Celle, Germany).

### 2.2. Methods

#### 2.2.1. Production and Characterization of Nile Red-Loaded Vaseline

Nile red-loaded Vaseline (V-NR) was obtained by dissolving NR in heated Vaseline (45 °C—heated on a water bath). After complete dissolution of NR in Vaseline, the mixture was cooled down while being stirred in a mortar with a pestle. After cooling, the formulation was filled into falcon tubes and stored at room temperature until further use. In order to obtain detailed information on the microstructure of the Vaseline and to ensure the complete dissolution of the dye within the formulations throughout the whole study, the formulation obtained was characterized by light microscopy (Olympus BX53, Olympus Corporation, Shinjuku, Japan), equipped with an Olympus SC50 CMOS color camera (Olympus soft images solution GmbH, Hamburg, Germany), and by inverted epifluorescence microscopy (Olympus CKX53), equipped with an Olympus DP22 color camera, (Olympus Life Science Solutions GmbH, Hamburg, Germany).

#### 2.2.2. Determination of Dermal Penetration Efficacy

**Figure 1:**
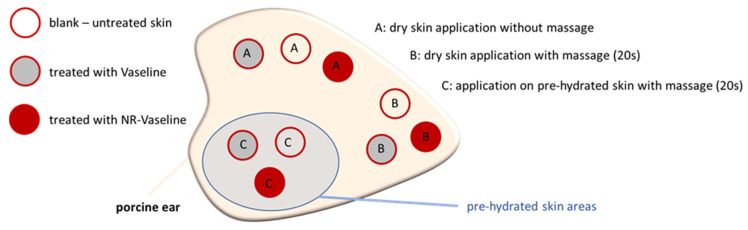
Experimental design for the determination of the dermal penetration efficacy. Experiments were performed in triplicate, i.e., on three different, independent ears.

The dermal penetration efficacy was determined on the ex vivo porcine ear model. The model uses non-modified skin that is still in its original physiological condition, i.e., it is still connected to the cartilage and thus remains in its original skin tension without any manipulation. Therefore, the model is considered as able to provide highly sensitive and sensible penetration data with high ex vivo/in vivo correlation [6,7,8,9]. For the experiments, fresh porcine ears were obtained from a local slaughterhouse and were used for the tests within 6 h after the slaughter. Prior to the tests, the ears were washed with lukewarm water and dabbed dry with paper towels. Intact skin areas without visible scratches or wounds were selected and marked. Hair within these areas was cut short (1–3 mm) and the integrity of the skin was ensured by assessing the transepidermal water loss (TEWL) within these areas with a Tewameter^®^ TM 300 (Courage + Khazaka electronic GmbH, Köln, Germany). Skin areas with TEWL values > 12 g/m^2^/h were considered to possess a disrupted skin barrier and were excluded from the study. Hydration of the skin was performed by placing a water-soaked paper tissue on top of the selected skin areas for 15 min. After 15 min, the tissue was removed and water on the skin surface was carefully dabbed off with a dry paper tissue. V-NR was applied in infinite dose setup (>10 mg/cm^2^) onto dry skin with and without massage (finger massage with saturated glove for 20 s) and onto the pre-hydrated skin with massage (Figure 1).

The penetration time was 3 h at 32 °C in an oven, with a relative humidity of about 65%. After incubation, the formulations were carefully removed from the skin with a soft tissue, and punch biopsies (Ø 12 mm) were taken from each sample. The obtained skin biopsies were immediately embedded in Tissue-Tek^®^ (Sakura Finetek Europe B.V., Alphen aan den Rijn, The Netherlands) and frozen until further use. Untreated skin and skin treated with pure Vaseline served as control (Figure 1). Each formulation was tested in triplicate and on different, independent ears.

The punch biopsies were vertically cut into 20 µm slices (cryomicrotome Mod. 2700, Reichert-Jung, Nußloch, Germany) and imaged with an inverted epifluorescence microscope (Olympus CKX53, equipped with an Olympus DP22 color camera, Olympus Life Science Solutions GmbH, Hamburg, Germany). The light source was a 130 W U-HGLGPS illumination system (Olympus Deutschland GmbH, Hamburg, Germany), which was set to 50%. The exposure time was 50 ms and the filter selected for analysis of the skin sections was the DAPI HC filter block system (excitation filter: 540–560 nm (BP), dichroic mirror 570 nm, emission filter: starting at 580 nm (LP)). Images were taken at 200-fold magnification. From each biopsy, at least 12 skin cuts and 36 images were obtained, which resulted in a total of at least 108 images for each formulation tested (*n* = 3).

#### 2.2.3. Digital Image Processing

**Figure 2:**
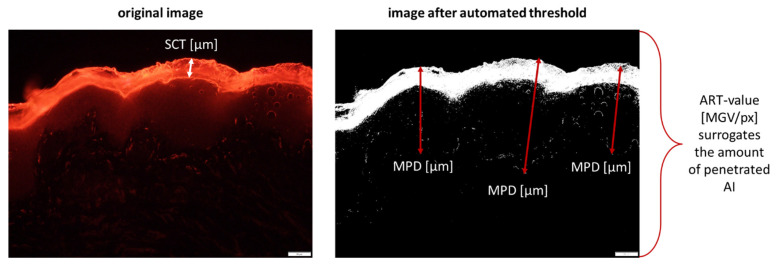
Overview of the parameters assessed with digital image analysis. SCT = stratum corneum thickness, MPD = mean penetration depth, ART = autofluorescence of image after RGB threshold.

The images obtained from epifluorescence microscopy were subjected to digital image analysis using ImageJ software [14,15,16]. The penetration efficacy was determined by assessing the amount of penetrated Nile red (AP-NR) semi-quantitatively and its mean penetration depth (MPD) into the skin by using a previously established protocol [17]. In addition, the stratum corneum thickness (SCT) was measured as a surrogate for skin hydration [6,7]. The AP and MPD were determined from the original images that were subjected to an automated RGB-threshold algorithm (Supplementary Materials S1) to subtract the autofluorescence of the skin (background, class II pixels) from the images [17]. The remaining light intensities in the images (foreground, class I pixels) correspond to the autofluorescence of the images after the RGB threshold (ART) and thus, were used as a semi-quantitative surrogate parameter to estimate the amount of penetrated AI surrogate [17]. The ART values were assessed as mean grey value per pixel (MGV/px) from each image [8,9,17]. The MPD was directly measured with the scale function of the software. The scale was set to correspond to 2.84 pixel/µm. Similar to the MPD, the SCT was assessed from the original images (Figure 2).

#### 2.2.4. Determination of Bio-Physical Skin Parameters

More detailed information about the influence of Vaseline, massage, and pre-hydration on the hydration of the skin was assessed by measuring the skin capacitance (Corneometer^®^ CM 825, Courage + Khazaka electronic GmbH, Köln, Germany) and the TEWL from the differently treated skin areas (Figure 1). In addition to Vaseline, the skin was also treated with liquid paraffin (mineral oil), which is a component of Vaseline. All formulations were applied with and without massage onto dry and pre-hydrated skin, respectively. The assessment of the bio-physical skin parameters is highly sensitive towards changes in temperature and humidity. Therefore, to avoid changes in TEWL and skin moisture due to changes in temperature and humidity that could occur if the ears are incubated in the oven at 32 °C with high humidity and then removed, the incubation time was set to 1 h at room temperature. Prior to the measurement, the formulations were gently wiped off with a soft paper tissue. After the measurements on the skin surface, the skin was tape stripped with duct tape (tesa extra Power^®^ Universaltape, tesa, Norderstedt, Germany) to remove the upper layers of the stratum corneum (SC) and to assess the capacitance and the TEWL in the deeper layers of the SC. The stripping procedure was performed once and removed about 12% of the SC (cf., Supplementary Materials S2).

#### 2.2.5. Statistical Analysis

Statistical analysis was performed with the JASP software (version 13.1.0.) [18]. Data were checked for normal distribution and variance homogeneity (Shapiro–Wilk test and Levene test) and were subjected to analysis of variance (one-way ANOVA or Kruskal–Wallis analysis of variance for the penetration data, repeated measures ANOVA for the corneometer and TEWL measurements). Adequate post hoc tests (Tukey, Games–Howell and Dunn) were performed to compare the mean values with each other. Selected data were also subjected to unpaired, one-tailed *t*-tests. These tests compare the mean values of two independent groups in only one direction and are thus considered capable of providing more power to detect whether a mean of a population is larger or smaller than the mean of another population [19]. The *p*-values < 0.05 were considered statistically significant.

## 3. Results

### 3.1. Production and Characterization of Nile Red-Loaded Vaseline

**Figure 3:**
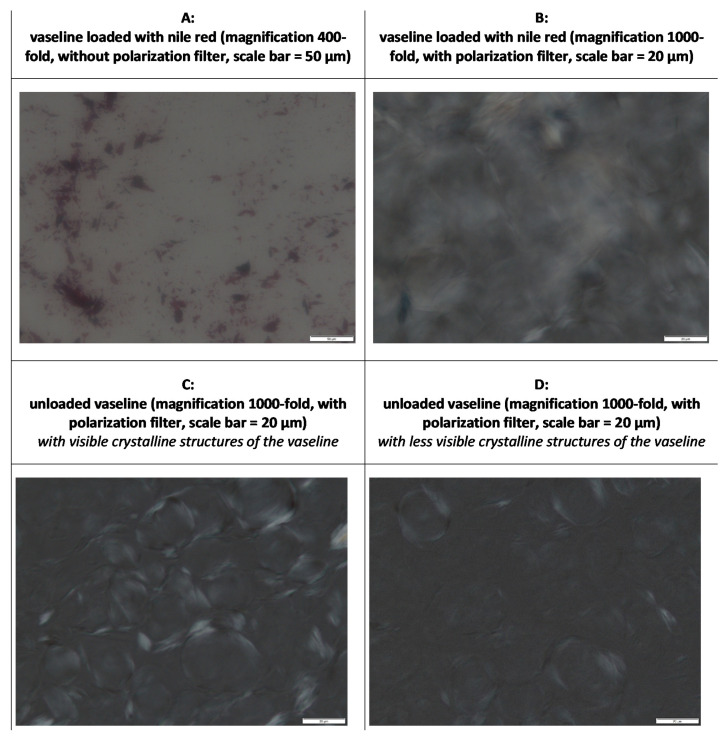
Microscopic images of Nile red-loaded Vaseline (**upper**) and unloaded Vaseline (**lower**)—for further explanations cf. text.

After loading, a homogenously red-colored semi-solid formulation was obtained. However, light microscopy showed that NR was not completely dissolved in the Vaseline, i.e., Nile red crystals were clearly detectable within the formulation (Figure 3A). Interestingly, it was observed that all the images obtained, especially the larger magnifications, appeared rather blurred and not sharp (Figure 3). Images with a polarization filter showed crystalline structures for both the loaded and unloaded Vaseline (Figure 3B–D). Hence, images confirm the presence of a three-dimensional crystalline network that consists of fiber-like crystals that immobilize the liquid hydrocarbons (Figure 3B,C). However, in some images, the crystalline structures appeared rather disperse and not connected to each other (Figure 3D). This might suggest that the Vaseline was also in the newly suggested structure by van Heugthen et al., i.e., where crystals form a network of lamellar stacks that trap the liquid fraction of petrolatum [20].

**Figure 4:**
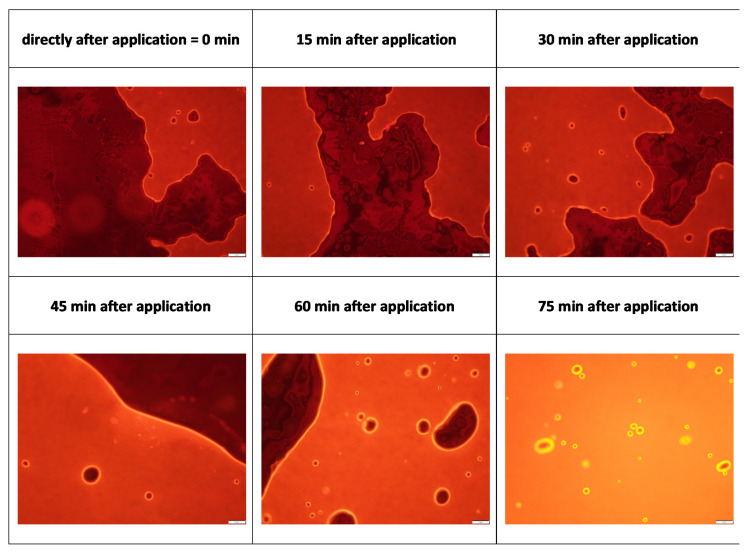
Images of Vaseline obtained from inverted epifluorescence microscopy. Images were taken at different time points (0–75 min). Images show the formation of a liquid film underneath the semi-solid Vaseline. The film appears immediately after application and increases over time until the whole interface between the microscope slide and Vaseline is filled with the film. By considering the microscope slide as a surrogate for the skin surface, images provide evidence that Vaseline undergoes syneresis after dermal application.

The microscopic analysis of the formulations with inverted epifluorescence microscopy revealed images that showed the appearance of a liquid film that enlarged over time (Figure 4). The inverted setup of the microscope allows for the observation of samples from the frog’s perspective, i.e., from underneath. Thus, the microscope slide acts as a surrogate for the skin and the images taken therefore show how a formulation behaves on the microscopic slide, i.e., the skin surrogate. In this light, one can see that Vaseline undergoes synereses, also known as “oil bleeding”, upon application onto the skin. This means that upon application onto the skin, the liquid hydrocarbons of the Vaseline were released from the crystalline network of the solid hydrocarbons and formed a film on top of the microscopic slight and/or the skin. The formation of the liquid film can also explain the blurred images obtained from light microscopy (Figure 3), because such a liquid film shifts the crystalline structures out of the focus of the lens, thus making the acquisition of sharp images difficult. To the best of our knowledge, the occurrence of syneresis after topical application of Vaseline on skin has not yet been described in the literature. However, the findings can be regarded as being of high relevance since the mineral oil film that is formed between Vaseline and the skin might affect various other parameters that are important for the dermal penetration of active compounds. This includes, for example, drug localization, drug diffusion and flux, concentration gradient of drug between vehicle and skin, etc. In addition, also the skin properties (for example, skin hydration or occlusive properties) might be influenced by this local syneresis effect.

### 3.2. Determination of Dermal Penetration Efficacy

**Figure 5:**
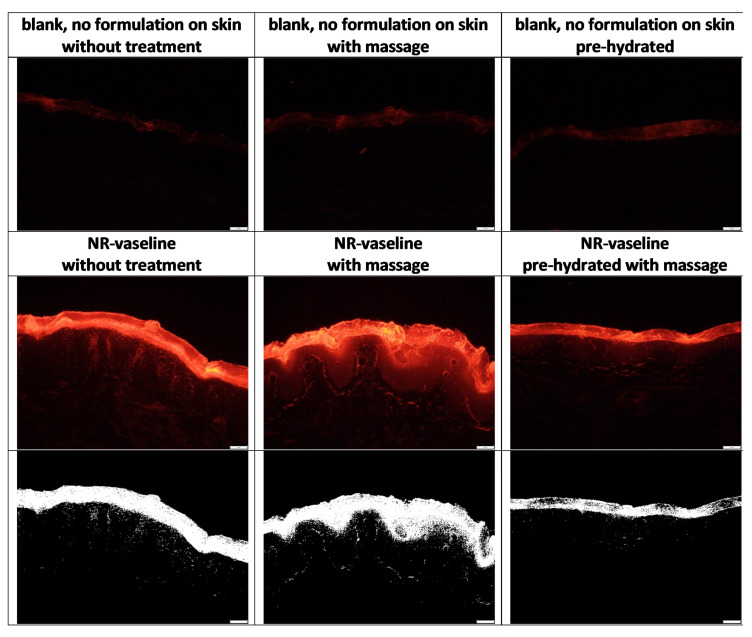
Images obtained from epifluorescence microscopy of the skin sections without Vaseline treatment (blank) and of the skin sections treated with Vaseline loaded with Nile red (middle row—original images, lower row—images after automated RGB Threshold). Scale bare = 50 µm.

**Figure 6:**
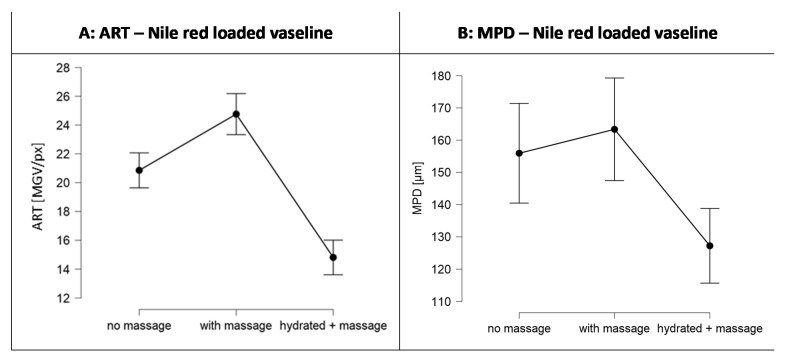
Dermal penetration efficacy of Nile red when loaded into Vaseline and applied onto skin without massage, with massage, and with massage on pre-hydrated skin. (**A**) ART-value (ART, MGV/px), a semi-quantitative parameter that estimates the amount of penetrated NR; (**B**) Mean penetration depth (MPD, µm).

The penetration efficacy was estimated from the visual inspection of the images obtained from epifluorescence microscopy (Figure 5) and by assessing the ART-value and the MPD (Figure 6). The images obtained from epifluorescence microscopy showed tremendous differences in the dermal penetration efficacy of Nile red on the differently treated skin areas. Application of the Nile red-loaded Vaseline on dry skin without massage allowed the Nile red to penetrate into the stratum corneum and to some extent into the epidermis and the viable dermis. Massage increased the ART value significantly by 19%, and the MPD was increased by 5% (*p* > 0.05). In contrast, application with massage on the hydrated skin decreased the ART value by 29% and the MPD by 18%. Both effects were significant (*p* < 0.001). The increased penetration efficacy of massage was expected. However, the decreased penetration efficacy that was observed when the formulation was applied onto pre-hydrated skin was not expected, because it was initially aimed at yielding a superposition by combining the three different skin penetration enhancing effects, i.e., occlusion, massage, and direct-skin hydration in one treatment.

### 3.3. Determination of Bio-Physical Skin Parameters

#### 3.3.1. Stratum Corneum Thickness

**Figure 7:**
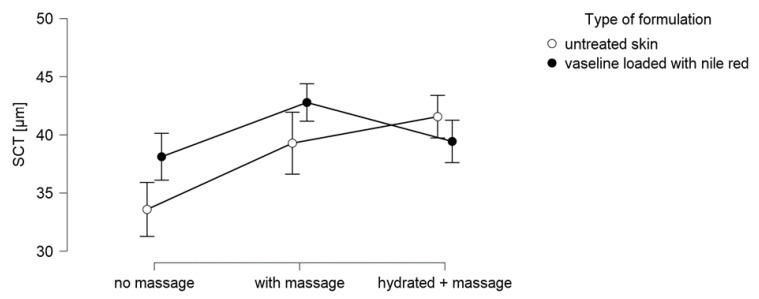
Influence of massage and skin hydration on the stratum corneum thickness (SCT).

The SCT of blank skin possessed a thickness of 34 µm (Figure 7). Massage of untreated skin increased the SCT significantly by 17%, and skin hydration caused an increase in SCT by 24%. The hydration of skin was expected to cause a swelling of the SC because it can be expected that the water penetrates into the SC. However, massage was not expected to cause any effect on the SCT. Nonetheless, the observed effect was significant (*p* < 0.01) and might therefore indicate that massage is also able to cause a deposition of water into the SC.

The application of Nile red-loaded Vaseline onto skin without massage caused a significant swelling of the SC (+13%) and proves that the application of Vaseline causes occlusion after dermal application. Hence, it forms a lipophilic film on top of the skin, which prevents the evaporation of water from the skin. The accumulation of water increases the water content in the SC and causes the SC to swell.

The application of Nile red-loaded Vaseline onto skin with massage also caused a significant increase in SCT when compared to the application without massage. However, the application onto hydrated skin resulted in a similar SCT as that with application of Vaseline onto dry skin without massage. Hence, the expected “super-hydration effect” could not be obtained by the combination of occlusion, massage, and pre-hydration of skin. The unexpected result needed further clarification. The water content on top of the skin and in deeper layers of the SC was therefore measured to understand the observed effect in more detail.

#### 3.3.2. Water Content

**Figure 8:**
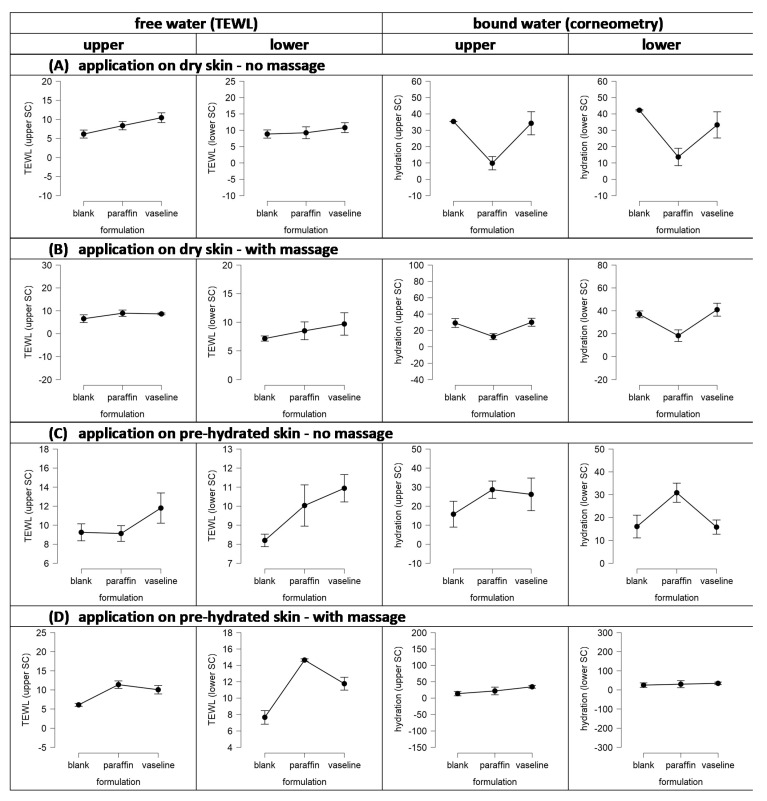
Influence of formulation, application, and skin condition on skin hydration. TEWL measurements were performed to assess the amount of free and unbound water. Corneometer measurements were performed to measure the capacitance of the skin tissue to assess the amount of water bound in the tissue. Measurements were taken from the skin surface (**upper**) and after tape stripping (**lower**) to assess the water content in the deeper layers of the stratum corneum (detailed explanations—cf. text).

The water content (free and bound water) of the skin surface and in the deeper layers of the SC was determined with TEWL and corneometer measurements. The Tewameter measures the amount of evaporating water from the skin per time and thus represents the amount of free and unbound water on or in the skin. Corneometry measures the capacitance of the surface and thus represents the water bound in the tissue. Measurements were taken from the upper surface and after tape stripping. With this, the water content (bound and unbound) was determined for the skin surface (upper) and for the deeper layers of the stratum corneum (lower). The influence of skin hydration and massage was tested by applying the formulations onto dry skin without (A) or with massage (B) or on pre-hydrated skin without (C) and with massage (D). The skin was treated with Vaseline or mineral oil (paraffin), while non-treated skin (no applied formulation) served as control. Data obtained are summarized in Figure 8 and Table 1.

The data show that the application of paraffin and Vaseline onto dry skin without massage significantly increases the free water on top of the skin. The effect was more pronounced for Vaseline (+70%) than for the paraffin (+36%). The trend was also seen for the lower SC, but it was not significant when compared to the untreated skin. With this, the data prove that Vaseline and paraffin cause an occlusion on the skin. Water from the skin cannot evaporate and thus accumulates on top of the SC, and—to some extent—also in the lower SC. The amount of bound water was decreased when the skin was treated with paraffin or Vaseline. The decrease was pronounced and significant for the paraffin-treated skin (−72%) and was small and non-significant for the Vaseline-treated skin (−3%). The measured decrease in skin corneometer values means that the capacitance of the tissue was decreased. A decrease in tissue capacitance can be considered to occur if a compound with low capacitance replaces the water on or in the tissue. As paraffin is a non-polar compound, its capacitance is zero. Hence, the decreased corneometer data indicate that paraffin penetrated into the SC, where it replaced the water. The small decreases in corneometer values after skin treatments with Vaseline might indicate that small portions of paraffin, e.g., due to local syneresis of the Vaseline after topical application, were also able to enter the skin.

**Figure 9:**
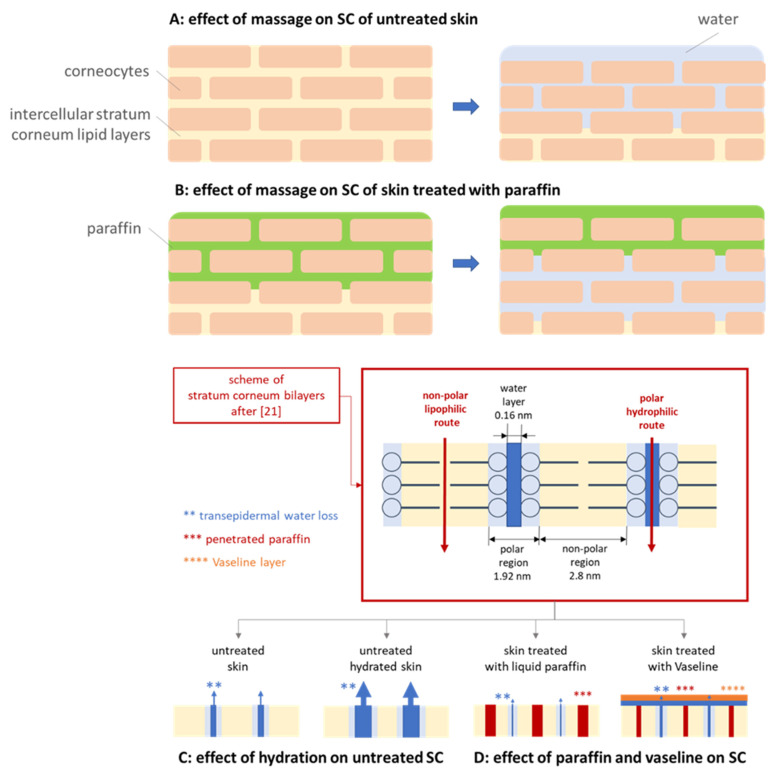
Scheme visualizing the effect of massage, occlusion, and direct hydration on the SC (explanation cf. text).

Massage of dry skin increased the free water on top of the skin (+7%), decreased the amount of free water in the lower layers of the SC (−19%), and reduced the content of bound water (approx. −15%). This indicates that the pressure during the massage causes the water from deeper layers in the skin to be squeezed out. The squeezed-out water forms a water layer on top of the skin and the water content in the lower layers of the SC is reduced (Figure 9A).

The application of paraffin onto dry skin with massage—when compared to the application of paraffin without massage—increased the free water on top of the skin (+7%), decreased the free water in the lower SC (−8%), and increased the bound water (approx. +30%). The observed effects indicate that the squeeze-out effect that was already observed for the blank skin was also obtained here. In addition, it can be considered that the applied pressure during massage forces the paraffin to penetrate deep into the skin. This creates two fronts of liquids with opposite directions of movement. The paraffin penetrates into the skin and the water moves towards the skin surface. The waterfront can be considered to prevent a deeper penetration of the paraffin, and the paraffin in the skin can be considered to counteract the movement of the water out of the skin (Figure 9B).

The application of Vaseline onto dry skin with massage—when compared to the application of Vaseline without massage—decreased the free water on top of the skin (−17%), decreased the free water in the lower SC (−10%), decreased the bound water in the upper SC (−13%), and increased the bound water in the lower SC (+23%). The results therefore show that the occlusion effect (free water on top of the skin) was reduced upon massage and became similar to the skin treated with paraffin and massage. Hence, it can be hypothesized that massage fosters the syneresis of Vaseline and increases the volume of mineral oil that penetrates the skin. The film on top of the skin is smaller, and therefore, the occlusive effect is reduced. In parallel, the pressure during massage causes the “squeeze-out effect” of the water and creates the formation of a waterfront in the lower layers of the SC, which prevents the penetration of the paraffin into deeper layers of the skin. The effect is less pronounced than for the paraffin, which indicates that the amount of penetrated paraffin from Vaseline is less and less deep when compared to pure paraffin.

Skin hydration increased the amount of free water on top of the skin (+50%), decreased the free water in the lower SC (−7%), and the amount of bound water (approx. 60%). Hence, diametral to the expectations, pre-hydration of the skin was found to cause a tremendous dehydrating effect of the skin. A possible reason is the localization of the water within the polar regions of the stratum corneum lipid bilayers [21], which might cause a widening of the “water pores”. The widening can be considered to increase the TEWL, and with this, more water can evaporate over time, eventually leading to the observed decrease in skin hydration (Figure 9C).

The application of paraffin onto hydrated skin yielded similarly high levels of free water on top of the skin as hydrated skin without treatment and as skin after application of paraffin on dry skin with massage. However, in contrast to the non-treated wet skin, that caused skin dehydration, application of paraffin on wet skin resulted in a strong increase in skin hydration. The free water in the lower SC was increased by about 10% and the water content of the bound water was increased by about 260%. The high levels of bound water in the SC indicate that pre-hydration of the skin allowed water to penetrate into the skin, where it formed a waterfront. Subsequent application of paraffin prevented the evaporation of water and at the same time the waterfront prevented the penetration of the paraffin (Figure 9B).

The treatment of wet skin with Vaseline resulted in a tremendous increase in the free water on top of the skin (+92%) when compared to untreated skin. When compared to the dry skin treated with Vaseline without massage, the effect was smaller (+13%). The free water in the lower SC was not affected and the content of bound water was decreased (approx. −40%). Hence, Vaseline could prevent the dehydration of the hydrated skin without treatment but could not increase the skin hydration as efficient as the paraffin. Data therefore suggest that the skin hydrating mechanism of paraffin is different to the mechanism of Vaseline. Maybe it can be assumed that the paraffin remains not totally on top of the skin but penetrates—at least in parts—within the non-polar regions of the intercellular stratum corneum lipid bilayers. The localization of the paraffin in the hydrophobic regions can then be considered to cause a swelling of the hydrophobic pathway whilst reducing the thickness of the hydrophilic regions. This reduces the “water pores” in the SC and leads to a decreased evaporation of water from the skin, thus, reducing the dehydration of the skin (Figure 9D).

Instead, Vaseline can be considered to form a protective layer on top of the skin. Therefore, water cannot evaporate from the surface and causes a higher water content of free water on top of the skin. The syneresis of vaseline causes some penetration of paraffin into the skin. However, the amount of penetrated paraffin is lower when compared to the treatment of the skin with pure liquid paraffin. Therefore, the prevention from dehydration, i.e., the decrease in the “water pores” is less pronounced, when compared to the paraffin treated skin. In addition, the penetration is not as deep, therefore the decreased dehydration is only seen for the upper SC layers but not for the deeper layers of the SC, where the dehydration was similar to the non-treated skin (Figure 9D). Massage of the hydrated untreated skin further accelerated the dehydration of the skin. The free water on top of the skin was similar to dry and non-treated skin. The free water in the lower SC was reduced by about 14% and the bound water was reduced by about 50%. Application of paraffin on wet skin with massage increased the free water by about 50% and the increase in bound water was about 220%. The effects observed can be explained by a superposition of the massage effect, i.e., water is squeezed out of the skin and mineral oil is forced to enter the skin. More penetrated paraffin replaces more water in the tissue and therefore the measured capacitance is lower when compared to the hydrated skin treated with paraffin without massage.

Application of vaseline on the hydrated skin with massage when compared to application of vaseline on dry skin without massage resulted in similar values, i.e., the free water and the content of bound water was not significantly different to the values for the dry skin treated with vaseline without massage. Hence, the diametral effects that were observed for massage, occlusion and dehydration cancelled out each other.

## 4. Discussion

Based on the data obtained it can be concluded that the expected super-hydration effect by applying vaseline on hydrated skin with massage to improve the dermal penetration efficacy was not observed. Instead, it was found that penetration of the lipophilic AI surrogate was hampered by this procedure. Hence, improved dermal drug delivery cannot be achieved by applying drug loaded vaseline on hydrated skin with massage. Instead, it can be concluded that drug loaded Vaseline should be applied on rather dry skin, where massage can be used to further improve the penetration results.

Despite these findings with important practical relevance, of course it is also interesting to understand the underlying mechanisms for the observed effects. After reconsideration of the results obtained it was hypothesized that the observed reduced penetration of Nile red from the Vaseline when applied on wet skin with massage (cf. Figure 6) is caused by the formation of an aqueous layer on top of the skin. The aqueous layer acts as a water barrier not only for the paraffin but also for lipophilic AI surrogate Nile red. Hence, the hydrophilic barrier formed, hampers the penetration of the lipophilic dye.

The increased penetration efficacy, and especially the increased penetration depth when applied on dry skin with massage (cf. Figure 6), cannot be explained by this theory, because the squeezed in water from deeper layers should at least cause a reduced penetration depth of Nile red when compared to the unmassaged, dry skin. Therefore, as an alternative, possible explanation for the increased penetration efficacy of Nile red after massage on dry skin, the penetration of the mineral oil after syneresis into the skin needs to be considered. The penetration of paraffin into the SC was confirmed by the data obtained from the biophysical skin properties (cf. Section 3.3.2) and also previous studies already confirmed the penetration of paraffin into the skin [11,12,22,23]. Massage was shown to foster local syneresis of Vaseline and to increase the penetration of the expulsed mineral oil into the skin. According to Neubert and Wepf, active compounds might penetrate via the hydrophilic pathway of the skin and via the hydrophobic pathway [21]. Therefore, in case of the penetrated non-polar mineral oil and/or mineral waxes, it can be assumed that they penetrate via the hydrophobic pathway (Figure 9D).

Based on the images obtained from fluorescence microscopy (Figure 4), it can be seen that the mineral oil acts as a solvent for the Nile red. Therefore, it appears reasonable that the AI-surrogate being dissolved in the mineral oil penetrates the skin together with the hydrocarbons (paraffin) and will not be left behind on top of the skin. Hence, it can be hypothesized that the Nile red enters the skin not only via passive diffusion but also via convection, i.e., via a solvent drag mechanism. Drug uptake via convection and solvent drag mechanisms are well described effects for oral application but were so far very rarely proposed, e.g., only for ethanolic solvents, for dermal application [24,25,26].

**Figure 10:**
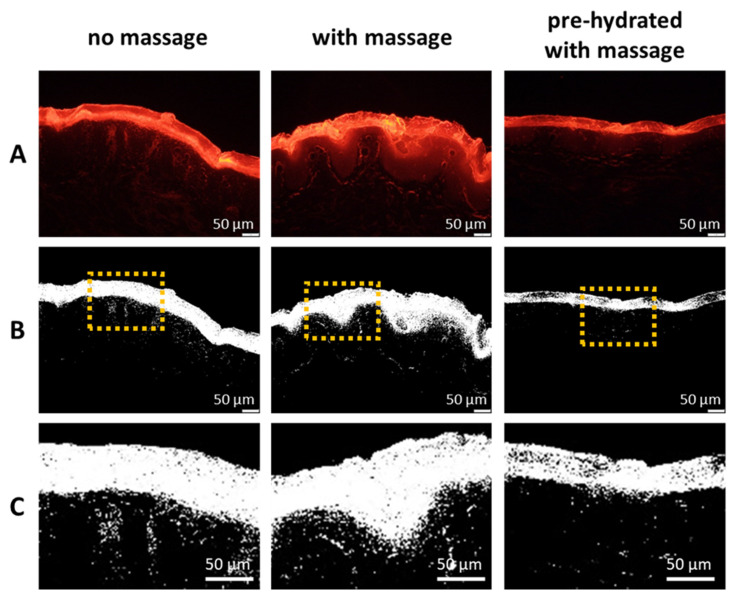
Influence of application, formulations, and skin condition on skin hydration and penetration efficacy of Nile red. (**A**): original images of skin sections obtained from epifluorescence microscopy, (**B**): images from (**A**) after digital RGB-threshold, (**C**): digital zooms of the dashed image sections from (**B**).

The assumption that Nile red enters the skin via a solvent drag mechanism together with the mineral oil in which it is dissolved is underlined by the different penetration patterns observed from the skin biopsies treated with Nile red loaded Vaseline with and without massage (Figure 5—lower, Figure 10). When the Nile red loaded Vaseline was applied on wet skin with massage, the Nile red is mainly located in the SC and almost no penetration into the epidermis and deeper layers of the skin is visible. This can be explained by the formation of a “waterfront” on top of the skin that is created during the massage. The waterfront hampers the penetration of the lipophilic dye. If a deeper penetration is detected (Figure 9B,C), the penetration pattern is characterized by single spots that penetrate individually into the skin. In contrast, “reddish waves” are clearly visible in the skin when the Nile red was applied on dry skin with massage. The waves seem to form a rim at the basal layer of the epidermis. After the rim (corresponding to the basal layer of the epidermis), the penetration of Nile red appears similar to the penetration pattern seen for the hydrated skin after the Nile red passed the stratum corneum. Hence, for the dry and massaged skin, it seems as the Nile red uses a “lift” until the end of the epidermis. The lift seems to be not present in the hydrated skin and only to a small extend in the dry skin were the Nile red loaded Vaseline was applied without massage (Figure 10). The “lift” can be considered to be the penetrated mineral oil, which’s penetration seems to stop at the end of the basal layer.

**Figure 11:**
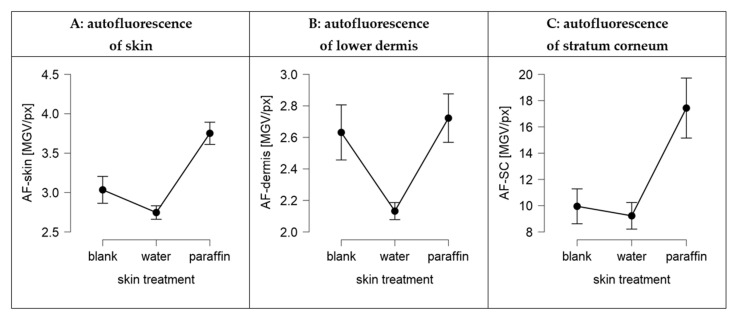
Changes in autofluorescence of skin sections upon application of paraffin or water on skin with massage in comparison to untreated massaged skin (blank). (**A**): mean autofluorescence of complete image, (**B**): mean autofluorescence of lower viable dermis of stratum corneum, (**C**): mean autofluorescence of stratum corneum (cf., Supplementary Materials S3).

**Figure 12:**
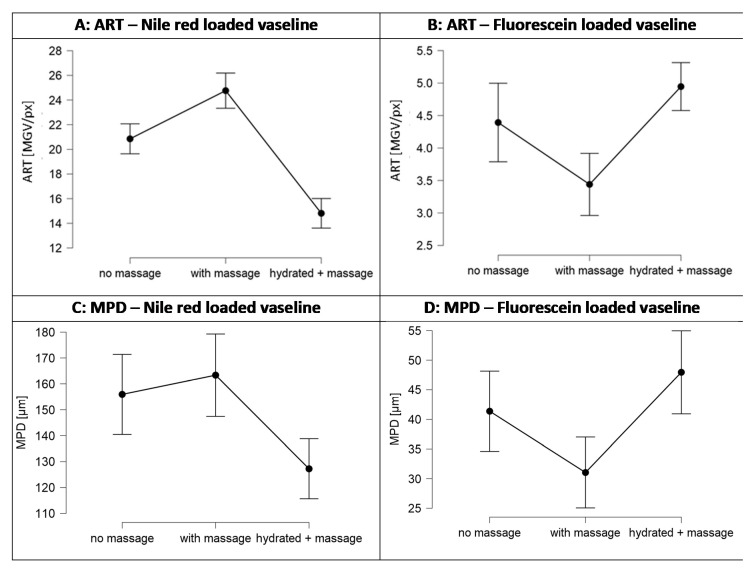
Comparison of influence of massage and skin hydration on the penetration efficacy of Nile red ((**left**), surrogate for hydrophobic AI—cf. Figure 6) and fluorescein ((**right**), surrogate for hydrophilic AI). Explanations cf. text.

The proposed penetration of paraffin into the skin and the solvent drag and waterfront hypotheses were proven in the next steps of the study. First, the penetration of paraffin and also a possible penetration of water into the skin was investigated by applying pure paraffin and pure water without any dye on skin and by comparing the changes in the skin autofluorescence between untreated and treated skin. Secondly, the solvent drag and waterfront hypotheses were proven by adding 0.003% (*w*/*w*) of a water-soluble, fluorescent dye (sodium-fluorescein) as a surrogate for a hydrophilic AI to Vaseline. Skin penetration experiments were performed similarly to those performed for the Nile red loaded Vaseline, i.e., on skin with and without massage and on pre-hydrated skin with massage (Figure 1, Figure 10, Figure 11 and Figure 12).

Both, the application of pure water and the application of pure paraffin resulted in significant changes in the autofluorescence of the skin (Figure 11). Water reduced the autofluorescence of the skin and paraffin increased it (Figure 11A). A more detailed analysis of the data revealed that the changes in the autofluorescence of the skin upon skin treatment with water were more pronounced in the deeper layers of the skin (Figure 11B) and less pronounced in the stratum corneum (Figure 11C). In contrast, changes in skin autofluorescence upon treatment with paraffin were less pronounced in the deeper layers of the dermis and most pronounced in the stratum corneum. Data therefore indicate that both, water and paraffin, penetrate into the skin. Water has no fluorescent properties and thus—once it penetrates into the skin tissue—decreases the autofluorescence of the skin tissue. Paraffin was found to possess a weak autofluorescence and thus increases the autofluorescence of the skin tissue [27]. The strong increase in the autofluorescence of the stratum corneum and the very limited increase of the autofluorescence in the lower dermis upon the skin treatment with paraffin indicates, that the paraffin enters the stratum corneum but cannot penetrate into deeper layers of the skin. The data therefore substantiate the findings of previous studies, that could already show a penetration of paraffin and other liquids into the skin [11,12,22,23,24,25,26,28]. Further, data of this study also demonstrate that water can enter the skin if applied on skin. The differences in autofluorescence between untreated skin and skin treated with water in the lower dermis indicate that water can penetrate into deeper layers of the skin. The limited differences between untreated and water treated SC indicate that the hydration of the stratum corneum is only limited. This finding is in line with the data obtained from the analysis of the biophysical skin parameters (cf. Section 3.3).

The solvent drag and waterfront hypotheses could also be confirmed, i.e., results obtained from the penetration studies with fluorescein loaded Vaseline prove the theory and confirm that the penetration of a hydrophilic AI is oppositely affected by skin hydration and syneresis of Vaseline due to massage. In fact, massage decreased the penetration of fluorescein and hydration increased it (Figure 12B,D). The decrease in penetration depth due to massage was about 25% and the increase due to application on pre-hydrated skin was about 15% for fluorescein. In contrast, massage increased the penetration of Nile red by about 15% and application on pre-hydrated skin decreased the penetration by about 25% (Figure 6 and Figure 12A,C).

Data therefore demonstrate that both, skin condition and type of application, can strongly affect the penetration efficacy of active compounds into the skin and confirm that an “all fits one” approach, i.e., massage and application on moist skin, is not appropriate, because—depending on the type of AI and the skin condition—it might have penetration enhancing or penetration reducing effects. The mechanism of an aqueous barrier between skin and topical formulation, which was found in this study, can be conclusively linked to the observations by H. Maibach and its co-workers, which stated: “occlusion is widely utilized to enhance the penetration of applied drugs in clinical practice; however, occlusion does not increase the percutaneous absorption of all chemicals” [29].

The results of this study fully confirm the observed effects by Maibach and co-workers and shed new light on the use of occlusive vehicles and skin massage for the improved dermal delivery of active compounds. In addition, data provide first evidence, that active compounds must not necessarily leave the vehicle to penetrate into the skin as they can directly penetrate along with the vehicle or parts of the vehicle in which they are dissolved. Based on the results obtained in this study it can be expected that many liquid ingredients of topical formulations might be able to penetrate into the skin. Despite paraffin, which was shown to penetrate the skin in this study, also other lipids (for example oils with low viscosity, e.g., medium chain triglycerides) can penetrate the skin [27,28] and thus allow for an enhanced penetration of lipophilic compounds which can be dissolved in these oils. The study could also demonstrate that water can penetrate into the skin. Thus, in theory, compounds being dissolved in the water might directly penetrate into the skin. These theories should be evaluated in more detail in future studies.

Nevertheless, data of this study already allow to conclude that penetration of active compounds from vehicles is not a static process that can be simply explained by Fick’s law of diffusion and by a simple consideration of distribution coefficients of the active compound between vehicle and skin. Dermal penetration of active compounds is rather a highly dynamic process that is composed of a complex interplay between skin, vehicle, type of application and changes in skin, vehicle and drug distribution after topical application. Based on the data so far it can be assumed that dermal penetration of active compounds from vehicles that are applied on the skin can be considered to be composed of at least three different mechanisms.

**Figure 13:**
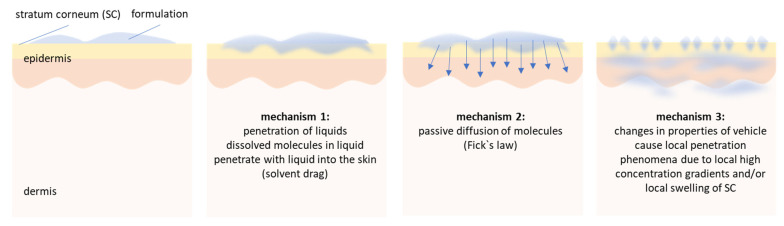
Proposed mechanisms of dermal penetration. 1st mechanism: penetration of low viscous liquids—active compounds penetrate via convection (solvent drag mechanisms) into the skin. 2nd mechanism: vehicle and skin are in equilibrium—AI penetrate via passive diffusion, 3rd mechanism: transformation of vehicle creates passive penetration phenomena—one example is the particle assisted penetration enhancement, i.e., the formation of aqueous menisci below particulate materials that increase the penetration of AI being dissolved in the liquid of the menisci [6]. It is likely that the different mechanisms occur not step-wise but superimpose each other.

The first mechanism involves the expulsion of low viscous liquids, e.g., water or oils, from the vehicle and their subsequent penetration into the skin. Active compounds that are dissolved in these liquids can enter the skin via convection, i.e., a solvent drag mechanism either via the polar or non-polar route. The second mechanism is the classical penetration pathway, i.e., passive diffusion of the AI from the vehicle into the skin. The third mechanism involves changes in skin properties and/or the vehicle that can influence and modify the dermal penetration of active compounds over time. For example, the penetration of the liquids into the skin, the non-penetrated formulation on top of the skin and also mechanical skin treatments (e.g., massage) can modify the skin properties and thus can alter the passive diffusion of the AI. Finally, when the formulation on top of the skin dries out, other phenomena might start to modulate the penetration efficacy. Possible changes include coalescence of oil droplets in emulsions and/or—in case the formulation contains particulate materials—the formation of an aqueous meniscus [6]. The aqueous meniscus connects particles to the skin, causes a local swelling of the SC and fosters the penetration of active compounds that are dissolved in the liquid of the meniscus (Figure 13).

It can be assumed that a detailed and mechanistic understanding of these complex processes will allow for the development of topical products with tailor-made penetration profiles and optimal skin-caring properties at the same time. More research needs to be conducted to understand all these mechanisms, effects and possible interdependencies that orchestrate together the dermal penetration of active compounds in more detail.

## 5. Conclusions

Vaseline undergoes syneresis upon dermal application. Upon syneresis the mineral oil released penetrates into the skin and active compounds that are dissolved in the oil can enter the skin via a solvent drag mechanism. Massage changes the skin hydration. Due to the pressure the skin tissue is squeezed, and water is pressed out, which causes a formation of a waterfront. The localization of the waterfront depends on the initial hydration state of the SC. High SC hydration causes the formation of the waterfront in the upper layers of the SC and on top of the SC. Lower skin hydration causes the formation in lower SC regions. The waterfront reduces the penetration of lipophilic chemicals. Oppositely, it can foster the penetration efficacy for hydrophilic compounds. Results of the study therefore prove that the often-proposed model of passive diffusion might be too simplistic to explain and forecast the dermal penetration efficacy of active compounds for different formulations. More effects, i.e., skin hydration, skin treatment and the penetration of excipients from the vehicle that can modify the SC properties must also be considered.

## Figures and Tables

**Table 1:** Influence of formulation, application, and skin condition on skin hydration. Results are expressed as relative values, i.e., the values obtained from the untreated skin were used as a benchmark control and were set to 100%. The values obtained for the other skin areas were related to these values and are presented as relative TEWL (%) and capacitance (%) ± relative standard deviation (RSD).

Formulation	Application	Relative TEWL (%)		Relative Capacitance (%)	
		Upper SC ± RSD	Lower SC ± RSD	Upper SC ± RSD	Lower SC ± RSD
**blank**	**no massage**	**100.0** ± 24.1	**100.0** ± 20.0	**100.0** ± 0.2	**100.0** ± 1.4
	**massage (m)**	**106.5** ± 36.7	**80.8** ± 8.9	**82.2** ± 26.3	**87.3** ± 11.4
	**hydration**	**150.4** ± 19.3	**92.7** ± 8.0	**44.6** ± 85.9	**38.0** ± 62.0
	**hydration + m**	**99.0** ± 13.1	**86.4** ± 21.4	**38.7** ± 99.1	**59.4** ± 93.4
**paraffin**	**no massage**	**135.8** ± 22.6	**104.3** ± 34.1	**27.8** ± 71.7	**32.3** ± 67.5
	**massage (m)**	**145.5** ± 30.9	**96.0** ± 36.7	**34.9** ± 60.6	**43.2** ± 55.8
	**hydration**	**148.4** ± 22.2	**113.4** ± 26.5	**80.9** ± 38.9	**73.0** ± 33.3
	**hydration + m**	**185.0** ± 12.1	**165.5** ± 1.4	**61.5** ± 76.2	**71.1** ± 87.1
**Vaseline**	**no massage**	**169.8** ± 27.5	**122.0** ± 31.4	**96.9** ± 46.2	**78.7** ± 53.8
	**massage (m)**	**140.7** ± 7.9	**109.6** ± 35.0	**84.7** ± 28.4	**96.9** ± 23.8
	**hydration**	**191.9** ± 30.1	**123.6** ± 14.7	**74.0** ± 73.2	**37.4** ± 43.9
	**hydration + m**	**163.4** ± 24.6	**132.9** ± 14.9	**97.6** ± 27.8	**81.4** ± 43.8

## Data Availability

Data is contained within the article or supplementary material.

## Supplementary Materials

The following are available online at https://www.mdpi.com/article/10.3390/pharmaceutics13122190/s1, Supplementary S1: RGB-Macro used for each image to discriminate class I from class II pixel. Figure S1: Influence of tape stripping with duct tape on the SCT. Figure S2: Scheme for the assessment of the different penetration data. Explanation cf. text.
