# Supplementary material for: Influence of Massage and Skin Hydration on Dermal Penetration Efficacy of Nile Red from Petroleum Jelly—An Unexpected Outcome
Source: Pharmaceutics. 2021 Dec 18;13(12):2190. doi: 10.3390/pharmaceutics13122190 (PMC8705314; doi:10.3390/pharmaceutics13122190)
Supplement: Supplementary file 1 [file pharmaceutics-13-02190-s001.zip › pharmaceutics-1466734-supplementary.pdf]

pharmaceutics

MDPI

Supplementary Materials: Influence of Massage and Skin Hydration on Dermal Penetration Efficacy of Nile Red from Petroleum Jelly—An Unexpected Outcome

Vasudha Kaushik, Yameera Ganashalingam, Robert Schesny, Christian Raab, Soma Sengupta and Cornelia M. Keck

Supplementary information

Supplementary material S1.

RGB-Macro used for each image to discriminate class I from class II pixels.

```
// Color Thresholder 1.53a
// Autogenerated macro, single images only!
min=newArray(3);
max=newArray(3);
filter=newArray(3);
a=getTitle();
run("RGB Stack");
run("Convert Stack to Images");
selectWindow("Red");
rename("0");
selectWindow("Green");
rename("1");
selectWindow("Blue");
rename("2");
min[0]=33;
max[0]=255;
filter[0]="pass";
min[1]=6;
max[1]=255;
filter[1]="pass";
min[2]=15;
max[2]=255;
filter[2]="pass";
for (i=0;i<3;i++){
  selectWindow(""+i);
  setThreshold(min[i], max[i]);
  run("Convert to Mask");
  if (filter[i]=="stop")    run("Invert");
}
imageCalculator("AND create", "0","1");
```

```
imageCalculator("AND create", "Result of 0","2");
for (i=0;i<3;i++){
   selectWindow(""+i);
   close();
}
selectWindow("Result of 0");
close();
selectWindow("Result of Result of 0");
rename(a);
// Colour Thresholding-------------
run „invert“
```

Supplementary material S2.

The decrease in SC thickness due to tape stripping with duct tape was determined by comparing the SCT of non-stripped and stripped skin. For this skin biopsies were obtained from non-stripped and stripped skin aeras. The biopsies were treated as described in 2.2.2 and the SCT was determined as described in 2.2.3. Results showed significant differences (student`s *t* test) between the stripped and non-stripped skin (Suppl. Figure S1) and the SCT of the stripped skin was 12% thinner than the non-stripped skin.

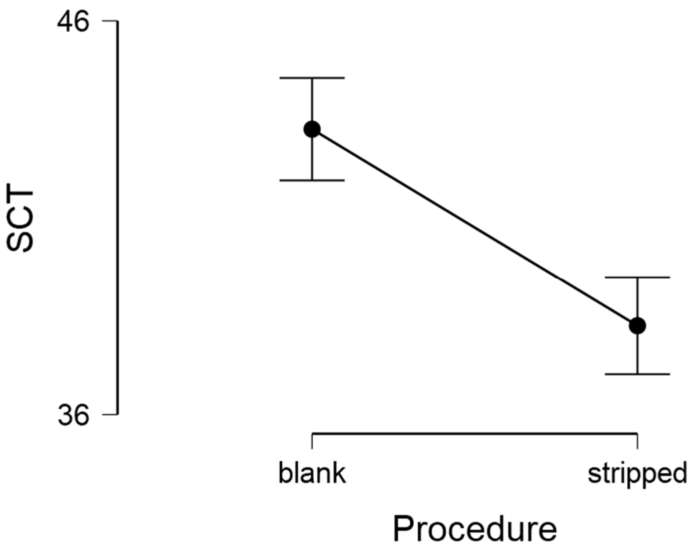

Figure S1. Influence of tape stripping with duct tape on the SCT.

Supplementary material S3.

The autofluorescence from the SC was assessed from the original images obtained from inverted epifluorescence microscopy and was measured from the SCT line (Suppl. Figure S2).

The autofluorescence of the dermis (AF dermis) was also determined from the original that were obtained from inverted epifluorescence microscopy. The parameter was assessed by applying a macro that selected a rectangle of the lower part of the images (Suppl. Figure S2). The rectangle was cut off and the ART-value within this section was determined. The macro applied for this was:

```
//setTool("rectangle");
makeRectangle(48, 1262, 1672, 158);
run("Crop");
run("Measure");
```

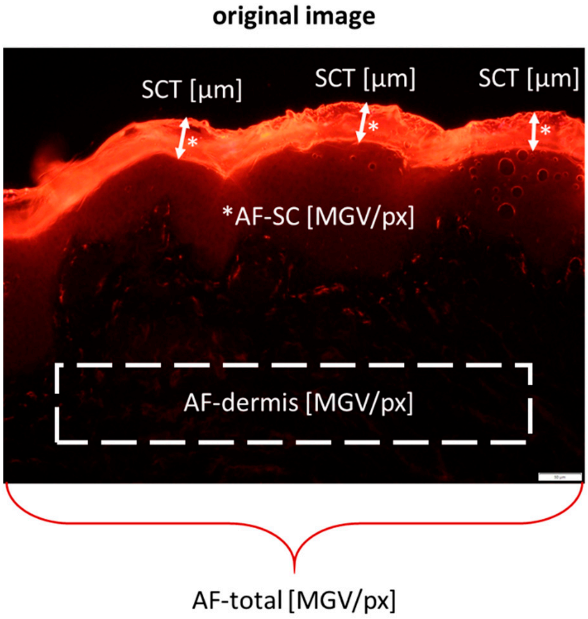

Figure S2. Scheme for the assessment of the different penetration data. SCT = stratum corneum thickness, * = autofluorescence of the stratum corneum. Explanation cf. text.
